# Factors influencing overweight children's commencement of and continuation in a resistance training program

**DOI:** 10.1186/1471-2458-10-709

**Published:** 2010-11-18

**Authors:** Melanie Pescud, Simone Pettigrew, Michael R McGuigan, Robert U Newton

**Affiliations:** 1 UWA Business School, University of Western Australia, 35 Stirling Highway, Crawley, 6009, Australia; 2 New Zealand Academy of Sport North Island, Millennium Institute of Sport & Health, Antares Place Mairangi Bay, 0632, New Zealand; 3 Vario Health Institute, Edith Cowan University, Joondalup Drive, Joondalup, 6027, Australia

## Abstract

**Background:**

In light of the child overweight and obesity problem in Australia, resistance training programs have been trialled as an innovative way of assisting children increase lean body mass and reduce body fat. The purpose of this study was to investigate the factors influencing overweight children's participation in a resistance training trial program.

**Method:**

Parent-child pairs who participated in the trial program were invited to take part in a follow-up individual interview to discuss their program experiences. In total, 22 semi-structured interviews were conducted with 11 parent-child pairs.

**Results:**

The factors found to be most relevant to program commencement among parents were a desire for their child to lose weight and gain confidence, the proximity of the venue, and no cost for participation. For children, the most relevant factors were the opportunity to build strength and improve fitness and having supportive parents who facilitated program initiation. The factors most relevant to continuation for parents were the quality of the program management, being able to stay for the sessions, the child's improved weight status, coordination, and confidence, and no cost for participation. Weight loss and improved confidence were also motivators for continuation among the children, along with pleasant social interaction with peers and trainers and ongoing parental support.

**Conclusion:**

Different factors variably influence program commencement and program continuation in both parents and children. This has important implications for future interventions that aim to successfully recruit and retain intervention participants.

## Background

Overweight and obesity in Australia constitute a widespread health problem that has been named a national priority by the National Preventative Health Taskforce [1]. Child overweight and obesity are particularly concerning because weight problems in childhood typically continue into adulthood [2,3]. One in four Australian children are currently overweight or obese [4], representing a substantial increase over the 1985 figures of 9% of boys and 10% of girls [5].

Overweight and obese children can experience numerous problems as a result of their weight status. These include neurological [6], gastroenterological [7,8], and orthopaedic complications [9]. Emotional problems are also common because overweight and obese children are more likely to be victims of bullying [10] and to experience low self-esteem [11]. They have been found to have higher anxiety and depression scores tracking from childhood into adulthood, and this is especially apparent in females [12]. Given these problems, it is important to develop effective interventions to prevent and treat child overweight and obesity. Such interventions require low attrition rates to achieve their potential, and it is this aspect of program development, within the context of resistance training programs, that was the focus of this study.

Exercise training, which encompasses both resistance and aerobic exercise, has been found to be beneficial in promoting healthy body composition and weight control for children aged six to 18 years [13,14]. While there have been concerns about negative impacts, it has been demonstrated that resistance training carried out in the appropriate manner is safe for children [15]. This form of training has been shown to be an especially appropriate form of exercise training for weight control in overweight and obese children because they are often stronger than their normal weight peers, making them more able to tolerate resistance work [16]. This is in contrast to aerobic exercise, which can be stressful on overweight and obese children's musculoskeletal systems as well as being particularly effortful for them [16]. Recent studies have demonstrated the efficacy of resistance training programs ranging from 6 to 12 weeks in duration for improving body composition in overweight and obese children [17-19]. Reflecting the success of these programs, the National Strength and Conditioning Association, the world authority in resistance training, has released a position statement noting the benefits of resistance training for young people [20]. Similarly, the United States Physical Activity Guidelines for Youth have been updated to include the recommendation of resistance training [21].

Resistance training programs can also have positive psychological outcomes for children. In particular, they have been shown to have a positive effect on physical self-perception in young people [22,23]. An example is the New Moves program that was introduced in the United States. New Moves was a trial obesity prevention program for overweight and at risk for overweight adolescent girls that incorporated exercise, social support, and nutritional components [24]. Four physical activity sessions were completed each week, with one of these sessions dedicated to strength training. Other activities included yoga, dance, and self-defence. Nutrition and social support sessions focused on healthy eating patterns and creating healthy body images. The program evaluation conducted with parents and children found high levels of satisfaction among both groups for all components. In particular, the girls felt that New Moves had led to an improvement in their activity levels and allowed them to become more accepting of their bodies.

Despite the benefits of physical activity, it can be difficult to encourage exercise initiation among overweight and obese children [25]. While peer pressure, being in poor health, and being unfit have been identified as motivating factors for obese adolescents to commence exercising behaviours [26], the factors affecting program commencement and continuation among younger children are not known.

Given the relative recency of resistance training programs for children and our poor understanding of the factors that influence participation in such programs, the purpose of the present study was to explore the barriers, motivators, and facilitators to overweight and obese children commencing and continuing a trial resistance training intervention program conducted in Perth, Western Australia. As children and their parents are likely to influence program participation [27], both groups were included in the study. The method used in the study is outlined below, followed by a discussion of the findings.

## Method

The children involved in the study participated in a resistance training program designed for overweight and obese children aged 6 to 13 years. Ethics clearance for the study was obtained from the Edith Cowan University Human Ethics Research Committee (ethics referral number 05-178). Participants were randomly assigned to one of three groups that varied in program duration: 8 weeks, 16 weeks, and 24 weeks. The physiological results of the study can be found elsewhere [28], but in summary the study demonstrated that resistance training made a significant improvement in body composition, power measures, and strength. For example, there was a mean improvement of 74% in lower body strength in the first eight weeks. At 24 weeks, the group that had continued training had improved their lower body strength by 135%. All groups trained three times per week for approximately 45 minutes for the duration of their programs. Participants could select from a range of session times (7 am - 8 am and 3.30 pm - 5 pm on weekdays and 9 am - 11 am on Saturdays). In addition to the aim of improved body composition, the program was designed to be enjoyable for children. This was achieved through manageable workloads, music being played during sessions, and efforts to encourage interaction between participants. As an example of the latter, the instructors made friendly conversation with multiple participants at a time to stimulate ongoing interaction among the children. The program was initially free, but a fee was imposed for continued participation upon completion of the research phase. Those choosing to continue with the program were charged an initial fee of $250 plus $8 per exercise session thereafter.

Participants were recruited into the program over a period of two months through local general practitioners and an advertisement in the local community newspaper. Sixty-three children were screened for inclusion, at which stage 13 were deemed ineligible. The resistance training component of the trial program was conducted by third-year undergraduate exercise science students at the University under the supervision of the chief investigators. Parents could either stay to observe their children during sessions or return to collect them later. In total, 31 families took up the opportunity to participate in the program. Six (19%) of the children completed the 8 week program, nine (29%) completed the 16 week program, and 16 (52%) completed the 24 week program. On average, 87% of sessions were attended by participants. The attrition rate by the end of the program was 87%, with only four of the 31 children who commenced the program enrolling in the subsequent fee-paying version.

### Evaluation sample

Eleven parent-child pairs participated in semi-structured individual interviews after completing the trial program. All families that started the program were invited to participate, including those who finished the program and those who withdrew. Of the participating children, one had completed the 8 week program, two had completed the 16 week program, and eight had completed the 24 week program. Table 1 provides the sample characteristics. Ten out of the 11 parents were mothers and one was a stepfather. Of the children, five were boys and six were girls. The interviewees were each reimbursed $AU50 for their time. At the time of the interviews, two children were normal weight, two children were overweight, and seven children were obese, as per Cole et al.'s international cut-offs for BMI [29].

**Table 1:** Sample characteristics

Pseudonym	Age	Parent	Intervention duration
Sarah	10	Step-father	8-week

John	8	Mother	16-week

Peter	11	Mother	16-week

Aaron	7	Mother	24-week

Lara	9	Mother	24-week

Michelle	9	Mother	24-week

Elizabeth	10	Mother	24-week

Larry	10	Mother	24-week

Sean	10	Mother	24-week

Tiffany	10	Mother	24-week

Cynthia	11	Mother	24-week

### Interviews

Parents and children were interviewed on the same day but separately to avoid contamination of responses. Informed consent was obtained from both the parents and the children. The interviewers had not previously met the interviewees and were not involved in program delivery, which encouraged the interviewees to give expansive and descriptive accounts of their experiences with the program. During the interviews, a wide range of topics was discussed including motivations to commence the program, perceptions of the program, and any problems that may have been experienced. Interviewees were also asked to reflect upon the positive and negative aspects of the program and to describe any barriers to their ongoing participation in similar programs in the future.

The interviews were digitally recorded with the interviewees' informed consent. The recordings were transcribed verbatim and imported into NVivo8 (a qualitative data analysis software program) for coding and analysis. Content codes were created to cover the topics listed in the interview guide and emergent concepts that were identified during analysis. Demographic characteristics of the interviewees were also captured.

## Results

All the adult interviewees and all but one of the child interviewees reported highly favourable experiences with the program. Only one child reported not enjoying the program, and this was attributed by both himself and his mother to his general dislike of exercise and strong preference to be sedentary. Both the parents and the children were able to discuss at length the factors that contributed to their decisions to commence and continue with the program. These factors are summarised in Table 2 and discussed below.

**Table 2:** Summary of factors influencing commencement and retention

Behaviour	Influencing Factors	Mainly Influencing
Commencement	Desire for weight loss	Parents

	Building strength and fitness	Children

	Improving confidence and self-esteem	Parents

	Parental commitment	Children

	Proximity of venue	Parents

	Low/no cost	Parents

Retention	Social interaction	Children

	Quality program management	Parents

	Parental commitment	Children

	Weight loss	Children and parents

	Improved confidence and self-esteem	Children and parents

	Improved coordination	Parents

	Low/no cost	Parents

	Parents welcome	Parents

### Factors Contributing to Program Commencement

When discussing the factors that led them to commence the program, the interviewees focused on anticipated outcomes and the proximity of the training venue in relation to the participants' homes. These two themes are outlined below.

#### Anticipated Outcomes

The parents reported that their primary motivation for enrolling their children in the resistance training program was for their children's health to improve through weight loss This was in accordance with the benefits stated in the advertisement for the program featured in the local newspaper, which included specific mention of the goal of reduced body fat. It was therefore not surprising that this factor emerged as the dominant motivator:

I think we could see that Sean was getting just a little bit of puppy fat and we thought we could strengthen him up. And also, he is a big boy so it would be nice to get his muscles moving a bit. (*Sean's mother, 24 weeks*)

I think it was that she wasn't doing very much in the way of exercise. She's a fairly solid sort of build, that sort of concerns her mother a little bit - partly because her father is a very solid build, quite overweight. So her mother is sort of worried about Sarah a bit, and so that was the motivation initially (that) came from her mother. And Sarah was happy to go along to it. I doubt very much that it was expressed in those terms to Sarah. (*Sarah's Stepfather, 8 weeks*)

Probably more the weight loss. Like I'd read they'd sort of said, you know, we're looking at children who are overweight and Tiffany was quite overweight for her height at the time. So it was like, "Oh great, this sounds like a program specifically tailored for Tiffany". (*Tiffany's Mother, 24 weeks*)

What were your expectations of the program? Basically for Larry to get healthy and to lose the extra weight he'd put on. (*Larry's Mother, 24 weeks*)

For some of the parents, an improvement in body weight was associated with enhanced self-esteem, which was another important motivator:

I think a lot of people were looking at improving self-esteem. Yeah, yeah it was good from that point of view, of self-confidence and self-esteem. (*Sean's Mother, 24 weeks*)

In terms of the children's motivations to commence the program, the boys were typically more interested than girls in the strength building and performance benefits of the program:

Well, I always wanted to get a bit faster because usually in the sports carnivals I always come last, so I wanted to become a bit faster. A bit of strength because my Dad always tried to push me down, but he always won and I wanted to beat him one time at least. (*Aaron, 24 weeks*)

My parents, I think they said, "Sean, would you like to do this program?"...They said, "You can go in and do some weights to build up some muscle", and I thought, "Oh yeah, I'll do that". (*Sean, 24 weeks*)

The girls tended to focus more on the general concept of health, which was described as being within a normal weight range and being fit.

It sounded interesting, and for health and fitness reasons. (*Tiffany, 24 weeks*)

Just because I wanted to lose weight and get healthier. (*Cynthia, 24 weeks*)

While most of the child interviewees were able to nominate reasons for their own interest in commencing the program, they acknowledged that initially it was their parents who wanted them to participate and made the decision to enrol them:

...I think it was more my parents wanted me to do it. (*Elizabeth, 24 weeks*)

My mum was the one who told me about it and I think there was an ad somewhere and she said, "Why don't you decide to do this? It will be good for you." *(Sarah, 8 weeks)*

#### Proximity

For those families living close by, a facilitator to enrol in the trial program was the close proximity of the facility to their place of residence. Most of the families lived near the University, (i.e. within five kilometres), and only few lived further afield (i.e. up to 25 kilometres). Proximity was perceived to be important because of the competing schedules of different family members that needed to be accommodated to allow attendance:

We were living only five minutes away, so we could drop her and then come back if we had things on. And some days a friend might drop her off if I was coming late home from work. So yeah, it worked in really well... We were lucky we were just living in (nearby suburb), so it was really good. (*Elizabeth's Mother, 24 weeks*)

As only those who participated in the trial program were interviewed, it was not possible to determine whether lack of proximity may have discouraged non-attendees from participating. However, it is likely that this played an important role given the need to accommodate siblings' and parents' other commitments and a lack of transportation options for some families.

### Factors Contributing to Program Continuation

The interviewees discussed several factors that encouraged program continuation, including the quality of the program management, social aspects, supportive parents, and physical and emotional improvements. There were also factors that appeared to act as barriers. For the parents, the primary issue was cost of the program once the study period had finished, and for the children there were the issues of physical exertion and ongoing motivation in the face of other commitments. Each of these factors is discussed below.

#### Program Management

The parents frequently mentioned that they were very impressed with the program coordinator. They especially appreciated his pleasant and welcoming nature and the way in which he ran the program.

(The man) who was running it, he was great. (*Elizabeth's Mother, 24 weeks*)

What he did was fantastic...he deserves a medal that guy. (*Lara's Mother, 24 weeks*)

The operational aspects of the program were considered to be well designed, such as in the wide range of training and body composition measurement appointment times that were available. These covered early morning, afternoon, and weekend options for the convenience of participants and their carers:

(The program manager) understood that only a few of us could come in the morning, so like when Tiffany had to do a re-scan he would make sure that whether it was him or someone else, that someone was there to do the scan in the morning. So that worked really well. (*Tiffany's Mother, 24 weeks*)

One mother discussed how the trainers assisted her son in continuing with the program by ensuring the timing of sessions was appropriate for his needs. The rest of the training groups wanted to change the session to a later time, but this mother was concerned about her son's motivation waning later in the day:

He would come at 4 pm and they wanted to change it to 4:30 pm. I said to them, "Look, if I go home, if he finishes school, goes home, gets changed, sits on the couch, eats something, then he's not going to want to go anywhere." And the guy that was actually training him said, "Oh it's all right, just bring him in at 4 pm and we'll just train him at 4 pm." (*Aaron's Mother, 24 weeks*)

When asked if there was anything that made attending the sessions difficult, the most common response was that on occasion they would arrive late because of traffic or that parents had to leave work early to make it on time. This was not seen as a major obstacle, largely because of the high level of perceived value of the program. Parents used a variety of strategies to overcome time constraints, such as choosing early morning rather than afternoon sessions and sharing transportation with other families in the program.

#### Social Aspects

The social aspects of the trial program were generally perceived to be one of the most positive elements of the experience. This had not been anticipated, but was found to be an important motivator for ongoing participation. Most of the parents and children explicitly mentioned the development of friendships among the children and between the children and their trainers. The friendships were not maintained once the program was completed, but for the duration of the program they were an important reason to continue. Friendships were particularly valued by the girls:

So that was another reason for me to go, because I knew I would be catching up with (friends), whereas I wouldn't normally see them... Sometimes when I said, "Oh, I really don't want to go this week", and I knew I should because it was a research program for the students here. And I did enjoy it a lot, and I knew it would be fun, and I'd always end up having a good time. (*Sarah, 8 weeks*)

I thought it was really good. I mean like, at the beginning I thought I was just going to do it to lose a bit of weight, but in the end I really enjoyed it because I made friends and yeah, I also lost weight. (*Cynthia, 24 weeks*)

Some of the parents mentioned that while their children did not make long-lasting friends with their peers during the sessions, they still enjoyed the social aspects of interacting with both the other children and the trainers. In many cases the trainers were not just seen as the children's teachers, but also as friends and mentors. This appeared to contribute to the children's enjoyment and encouraged their continued participation in the program.

No, he didn't make friends with any of the kids. He more made friends with the students who were running the program. And then we used to see them at the shops and things like that and say, "Hello", so that was good...They used to have a laugh, and a joke, and they liked that. (*Sean's Mother, 24 weeks*)

The trainers were excellent with the kids. They were really, really kid orientated. So they made it lots of fun while they were doing all the work. (*Elizabeth's Mother, 24 weeks*)

Researcher: What were your favourite parts of the program? I suppose the actual students that were running it, they were really friendly. As you'd wait there to start the program they'd always have a big smile, "Hello, come on in and we'll start up." ...They helped us enjoy it and they were really good. (*Sarah, 8 weeks*)

#### Parental Support

Parents were found to play two primary roles in facilitating their children's ongoing involvement in the trial program. First, as noted above, they were instrumental in transporting children to and from the sessions. This was often a logistical problem that required purposeful planning by parents to overcome. The ability of parents to stay for the training sessions was appreciated by some parents because it reduced total travel time. It also allowed them to observe their children's progress and learn more about how the exercises were performed.

I tended to sit in the room and watch so I could try and see how they were telling her how to do the exercises properly, and the stretches. (*Tiffany's Mother, 24 weeks*)

Second, in a small number of instances it was reported that parents had to actively encourage their children to attend, particularly if they were tired or had numerous other commitments at that point in time. In the case of the one unwilling child, this encouragement took the form of bribery to ensure he completed the program:

Mum blackmailed me with things like computer games and a computer and stuff like that. (*Larry, 24 weeks*)

#### Physical and Emotional Improvements

Some of the children reported enjoying the new sense of ability derived from the exercises. This became a source of ongoing motivation to continue with the program:

I actually enjoyed it, I thought it was good. I enjoyed the exercises and knowing what I can and can't do, and being able to...I enjoyed challenging myself and being able to know more about myself and what I am capable of, and yeah, it was really good. (*Sarah, 8 weeks*)

Several of the parents and a few of the children mentioned that evident weight loss was a major motivator for continued participation in the program. The body composition results that were given to the families on a regular basis were found to be useful in providing a tangible measure of weight loss, which motivated continued participation in the program.

She did lose quite a bit of weight at the time. (*Cynthia's Mother, 24 weeks*)

It was pretty good. It actually helped Liam to lose weight. (*Liam's Mother, 24 weeks*).

He did really well, like with the DEXA scans and all that sort of stuff, and like you know, he'd lost five percent body fat in the research program. (*Aaron's Mother, 24 weeks*)

If you're really like obese, like I was, you lose heaps of weight and you just feel good. (*Lara, 24 weeks*)

Many of the parents also noticed improvements in their children's confidence. They reported that by giving their children an opportunity to be good at something, the program increased their self-esteem:

I think confidence was a really big thing. She got more enthusiastic as she was doing the exercises and things like that. I think that confidence is very important to children that may be a little bit chubbier than others, you know. And it's important that they get more comfortable with their physical self. Once you start feeling better and stronger, well then you go on to more things. (*Cynthia's Mother, 24 weeks*)

He doesn't like to do team sport. He's not that good at team sports or anything like that. It's really repetitive and I just know that whenever we tried to get him to do something he wouldn't. He was like, "Oh no, I might not be good enough", and all this sort of stuff. So the reason for this was just to get him to do something, but it was an individual thing as well. So it was just him and he knew he could do it. Like once he started doing it, he knew he could do it and he didn't have to please anybody else. It was all about himself. (*Aaron's Mother, 24 weeks*)

Some of the children, especially the girls, were also aware that their self-perceptions altered during the program as a result of their growing capabilities and weight loss.

I enjoyed challenging myself and being able to know more about myself and what I am capable of. (*Sarah, 8 weeks*)

...you just feel good about yourself; you don't feel like different to others. (*Lara, 24 weeks*)

Improved coordination was described by some parents as an important further reason to ensure that their children continued with the trial program. This was particularly important for the parents of the girls, perhaps reflecting a social expectation for girls to be graceful [30].

She's not tremendously well coordinated and her coordination improved, and her overall confidence. (*Cynthia's Mother, 24 weeks*)

Her coordination has gotten better and her balance in general. (*Tiffany's Mother, 24 weeks*)

### Barriers

Three potential barriers to retention were identified. Numerous parents mentioned the cost associated with migrating to the new program once the research phase was completed as a substantial barrier to continuation. Many of the parents were low income earners who could not afford the new program regardless of its benefits for their children:

I would have loved to have kept doing the program, and I would probably be doing it now, I just can't afford to do it. (*Cynthia's Mother, 24 weeks*)

Yeah, she really enjoyed it, she was hoping there was going to be another one, but they said they had to pay for the next one. So yeah, we just couldn't afford it. (*Michelle's Mother, 24 weeks*)

I came along to all of the sessions that they had. I would have liked to have continued it, but I think after the initial training sessions they did offer it, but it had a fee. So, and that's sort of what's holding us back at the moment, because I can't afford it at this point in time. (*Peter's Mother, 16 weeks*)

It was all good except the only thing was there was nowhere for the kids to go and exercise in a similar program after it finished. I'm on a single income so I cannot afford to pay for Larry to do another program like this one. (*Larry's Mother, 24 weeks*)

This finding illustrates the substantial barrier of cost, suggesting that it should be a primary consideration in programs designed for less affluent families.

## Discussion

The findings indicate that different factors influence program commencement and program continuation. In this study, the children's parents provided the initial impetus for program commencement. Over the course of the program, in all but one case, the children's personal experiences fostered a desire to continue, bringing the parents' and children's preferences more closely in line. Interestingly, concerns about safety did not emerge as an influencing factor for program commencement or continuation, although it was an important factor in Faigenbaum's [15] review paper examining parental and child perceptions and experiences of resistance training programs.

Both parties especially appreciated the children's improvements in confidence and self-esteem, outcomes that have also been identified in other studies investigating the benefits of resistance training programs for children [22,23,31]. Most of the parents and a few of the children also specifically mentioned that the children lost weight as a result of participating in the program. The proximity of the program to their homes was a further important factor and points to the need for similar programs to be locally available. These outcomes illustrate that strategies employed to encourage initial participation may need to focus on parents, while efforts to minimise attrition may need to ensure both parents and children can see value in the program.

Having parents who are encouraging and regularly participate in physical activity themselves has been found to be important in influencing children to be more active [32,33]. The parents in the current study all strongly encouraged their children's participation in the program, and this was an important influence on both program commencement and continuation. Many of the children explicitly acknowledged that they had supportive and encouraging parents and that this was important in their continued participation in the program. However, emotional support was inadequate to ensure continued participation for most of the children because the introduction of fees after the free trial period constituted an insurmountable barrier for these families. This has important implications for program designers and managers who will need to consider program affordability for their targeted population. As child obesity is disproportionately prevalent in lower socioeconomic families [34,35], it seems likely that successful programs will need to be either low cost or free to encourage attendance.

The study findings also have considerable significance for governments trying to tackle child obesity, as currently in Australia there is no financial or medical support for children who are overweight or obese. The addition of Medicare (Australian national health system) items to address child obesity would seem imperative if the Australian Government is to address what is an increasingly expensive social and financial problem.

## Conclusion

The study findings demonstrate that different factors variably influence program commencement and program continuation in parents and children. This has important implications for future interventions that aim to successfully recruit and retain intervention participants.

## Strengths and Limitations

Given the qualitative nature of this study, it is not possible to determine the extent to which the findings are representative of the views of all the program participants and their parents. In particular, there is little representation of families from the 8 and 16 week programs. Also, the study did not explore the barriers for commencement that prevented other families from enrolling in the trial program in the first instance. Given the repetitive nature of resistance training programs, it is a further limitation of the study that the issue of boredom was not addressed with the interviewees. However, the interview data provide insight into numerous factors that can influence program commencement and continuation, providing program designers and managers with important information to consider in the development of future recruitment and retention strategies for resistance training programs.

## Competing interests

The authors declare that they have no competing interests.

## Authors' contributions

MRM, RUN, and SP participated in the design of the study. MP and SP conducted the interviews and carried out the thematic analysis of the transcripts. All authors assisted in drafting the manuscript and read and approved the final manuscript.

## Pre-publication history

The pre-publication history for this paper can be accessed here:

http://www.biomedcentral.com/1471-2458/10/709/prepub
